# Linked open data-based framework for automatic biomedical ontology generation

**DOI:** 10.1186/s12859-018-2339-3

**Published:** 2018-09-10

**Authors:** Mazen Alobaidi, Khalid Mahmood Malik, Susan Sabra

**Affiliations:** 1 Computer Science and Engineering Department, Oakland University, 2200 N. Squirrel Rd, Rochester, MI 48309 USA; 2 Micro Focus International plc, Troy, MI 48084 USA

**Keywords:** Semantic web, Ontology generation, Linked open data, Semantic enrichment

## Abstract

**Background:**

Fulfilling the vision of Semantic Web requires an accurate data model for organizing knowledge and sharing common understanding of the domain. Fitting this description, ontologies are the cornerstones of Semantic Web and can be used to solve many problems of clinical information and biomedical engineering, such as word sense disambiguation, semantic similarity, question answering, ontology alignment, etc. Manual construction of ontology is labor intensive and requires domain experts and ontology engineers. To downsize the labor-intensive nature of ontology generation and minimize the need for domain experts, we present a novel automated ontology generation framework, Linked Open Data approach for Automatic Biomedical Ontology Generation (LOD-ABOG), which is empowered by Linked Open Data (LOD). LOD-ABOG performs concept extraction using knowledge base mainly UMLS and LOD, along with Natural Language Processing (NLP) operations; and applies relation extraction using LOD, Breadth first Search (BSF) graph method, and Freepal repository patterns.

**Results:**

Our evaluation shows improved results in most of the tasks of ontology generation compared to those obtained by existing frameworks. We evaluated the performance of individual tasks (modules) of proposed framework using CDR and SemMedDB datasets. For concept extraction, evaluation shows an average F-measure of 58.12% for CDR corpus and 81.68% for SemMedDB; F-measure of 65.26% and 77.44% for biomedical taxonomic relation extraction using datasets of CDR and SemMedDB, respectively; and F-measure of 52.78% and 58.12% for biomedical non-taxonomic relation extraction using CDR corpus and SemMedDB, respectively. Additionally, the comparison with manually constructed baseline Alzheimer ontology shows F-measure of 72.48% in terms of concepts detection, 76.27% in relation extraction, and 83.28% in property extraction. Also, we compared our proposed framework with ontology-learning framework called “OntoGain” which shows that LOD-ABOG performs 14.76% better in terms of relation extraction.

**Conclusion:**

This paper has presented LOD-ABOG framework which shows that current LOD sources and technologies are a promising solution to automate the process of biomedical ontology generation and extract relations to a greater extent. In addition, unlike existing frameworks which require domain experts in ontology development process, the proposed approach requires involvement of them only for improvement purpose at the end of ontology life cycle.

## Background

In the era of Big Data and the immense volume of information and data available today on the web, there is an urgent need to revolutionize the way we model, organize, and refine that data. One way of modeling data is designing ontologies and using them to maximize the benefit of accessing and extracting valuable implicit and explicit knowledge from structured and unstructured data. Ontology is a vital piece in transforming the Web of documents to the Web of data [1]. The basic principle of ontology is representing data or facts in formal format using one of the primary ontology languages, namely, Resource Description Framework (RDF) [2], Resource Description Framework Schema (RDFs) [3], Web Ontology Language (OWL) [4], or Simple Knowledge Organization System (SKOS) [5].

Over the past decade, ontology generation has become one of the most revolutionary developments in many fields and the field of Bioinformatics. There are various approaches to create ontologies. These approaches include: rule-based & syntax analysis [6–11], syntactic patterns [12–16], dictionary-based [17] machine learning [18–24], and knowledge-based [25–27]. The rule-based approach involves a manually crafted set of rules formed to represent knowledge that decide what to do or conclude across various scenarios. Typically, it achieves a very high level of precision, but quite low recall. This approach is labor intensive, works for one specific domain, and is less scalable [10, 11]. On the other hand, syntactic pattern-based approach is well-studied in ontology engineering and has already been proven to be effective in ontology generation from unstructured text [12, 13]. Unlike the rule-based approach, this approach comprises a large number of crafted syntactic patterns. Therefore, it has high recall and low precision [14]. The crafted patterns are most likely broad and domain dependent. One of the most well-known lexico-syntactic pattern frameworks is Text2Onto [15]. Text2Onto combines machine learning approaches with basic linguistic approaches such as tokenization and part-of-speech (POS) tagging [16]. This approach suffers from inaccuracy and domain dependency. Naresh et al. [17] proposed a framework to build ontology from text that uses predefined dictionary. The drawbacks of their approach include labor cost to construct and maintenance of comprehensive dictionary. Finally, the resultant generated ontology was even manually created. Machine learning-based approaches use various supervised and unsupervised methods for automating ontology generation tasks. Studies in [18–22] present their proposed approaches for ontology generation based on supervised learning methods. In [18] Bundschus et al. focus on extracting relations among diseases, treatment, and genes using conditional random fields, while, in [19] Fortuna et al. use SVM active supervised learning method to extract domain concepts and instances. Cimiano et al. [20] investigate a supervised approach based on Formal Concept Analysis method combined with natural language processing to extract taxonomic relations from various data sources. Poesio et al. [21] proposed a supervised learning approach based on the kernel method that exploits exclusively shallow linguistic information. Huang et al. [22] proposed a supervised approach that uses predefine syntactic patterns and machine learning to detect relations between two entities from Wikipedia Texts. The primary drawback of these supervised machine learning based approaches is that they require huge volumes of training data, and manual labeling which is often time consuming, costly, and labor intensive. Therefore, few unsupervised approaches in [23, 24] were proposed: in [23] Legaz-García et al. use agglomerative clustering to construct concept hierarchies and generate formal specification output that complies with an OWL format by using ontology alignment while Missikoff et al. [24] proposed an unsupervised approach that combines a linguistic and statistics-based method to perform automated ontology generation tasks from texts.

Recently, some approaches that use knowledge-base to automate ontology construction have been proposed. For example, Harris et al. [24] use natural language processing and knowledge base, to construct ontological knowledge structure from raw text. The proposed approach uses a predefined dictionary of concepts to extract ‘disorder type’ concepts of ontological knowledge such as UMLS that might occur in the text. In addition, to extract the hierarchy relations, they use syntactic patterns to facilitate the extraction process. The drawbacks of their approach include labor cost to construct dictionary, domain specific, limited number of patterns. Another attempt using knowledge base approach was made by Cahyani et al. [25] to build domain ontology of Alzheimer using controlled vocabulary, and linked data patterns along with Alzheimer text corpus as an input. This study uses Text2Onto tools to identify concepts and relations and filters them using dictionary-based method. Furthermore, this work uses linked data patterns mapping to recognize the final concepts and relations candidates. This approach presents a few fundamental limitations: disease specific, requires predefine dictionary related to the domain of interest, and does not consider the semantic meaning of terms during concepts and relations extraction. Also, Qawasmeh et al. [27] proposed a semi-automated bootstrapping approach that involves manual text preprocessing and concept extraction along with usage of LOD to extract the relations, and instances of classes. The drawbacks of their approach include need of domain experts and involvement of significant manual labor during development process. Table 1 shows a comparison of proposed approach with existing knowledge-based approaches.

**Table 1:** A comparison of LOD-ABOG with existing knowledge base approaches

Modules	Approaches			
	Harris et al. (2015)	Cahyani et al. (2017)	Qawasmeh et al. (2018)	Proposed Approach (LOD-ABOG)
Text processing				
Methods	NLP	NLP	Manual	NLP
Concept Extraction				
Methods	*Dictionary lookup*, Statistical information	*Dictionary lookup*	Manual	*UMLS Mapping*, LOD
Evaluation	Accuracy 60% (domain independence), 90% domain specific	Accuracy 72% (represent concepts and relations)	Not available	recall 81.13%, precision 45.29%, F-measure 58.12%
Relation Extraction				
Methods	*Syntactic Patterns*	*Syntactic Patterns*	*LOD*	*Rule based*, *Syntactic Patterns, Semantic Enrichment, LOD, BSF*
Evaluation	Accuracy 31–67%	Accuracy 72% (represent concepts and relations)	Accuracy in range (15–50%)	Recall 63.82%, Precision 66.77%, F-measure 65.26%
Type of extracted data	List of concepts, relations between them, and synonyms	List of concepts, and relations between them	List of classes, relations between them, and instances of these class	OWL Ontology

Despite the ongoing efforts and many researches in the field of ontology building, many challenges still exist in the automation process of ontology generation from unstructured data [28, 29]. Such challenges include concepts discovery, taxonomic relationships extraction (that define a concept hierarchy), and non-taxonomic relationships. In general, ontologies are created manually and require availability of domain experts and ontology engineers familiar with the theory and practice of ontology construction. Once the ontology has been constructed, evolving knowledge and application requirements demand continuous maintenance efforts [30]. In addition, the dramatic increase in the volume of data over the last decade has made it virtually impossible to transform all existing data manually into knowledge under reasonable time constraints [31]. In this paper, we propose an automated framework called “Linked Open Data-Based Framework for Automatic Biomedical Ontology Generation” (LOD-ABOG) that resolves each of the aforementioned challenges at once; to overcome the high cost of the manual construction of a domain-specific ontology, transform large volume of data, achieve domain independency, and achieve high degree of domain coverage.

The proposed framework performs a hybrid approach using knowledge-base (UMLS) [32] and LOD [33] (Linked life Data [34, 35] BioPortal [36]), to accurately identify biomedical concepts; applies semantic enrichment in simple and concise way to enrich concepts by using LOD; uses Breadth-First search (BFS) [37] algorithm to navigate LOD repository and create high precise taxonomy and generates a well-defined ontology that fulfills W3C semantic web standards. In addition, the proposed framework was designed and implemented specifically for biomedical domains because it is built around the biomedical knowledge-bases (UMLS and LOD). Also, the concept detection module uses biomedical specific knowledge base-Unified Medical Language System (UMLS) for concept detection. However, it is possible to extend it for non-biomedical domain. Therefore, we will consider adding support for non-medical domain in future works.

This paper answers the following research questions. Whether LOD is sufficient to extract concepts, and relations between concepts from biomedical literature (e.g. Medline/PubMed)? What is the impact of using LOD along with traditional techniques like UMLS-based and Stanford API for concept extraction? Although, LOD could help to extract hierarchical relations, how can we affectively build non-hierarchical relations for resultant ontology? What is performance of proposed framework in terms of precision, recall and F-measure compared to one generated by automated OntoGain framework, and manually built ontology?

Our main contributions compared to existing knowledge-based approaches are as follows:

1. To address the weakness, and to improve the quality of the current automated and semi-automated approaches, our proposed framework integrates natural language processing and semantic enrichment to accurately detect concepts; uses semantic relatedness for concept disambiguation, applies graph search algorithm for triples mining, and employs semantic enrichment to detect relations between concepts. Another novel aspect of proposed framework is usage of Freepal: a large collection of patterns for relation extraction along with pattern matching algorithm, to enhance the extraction accuracy of non-taxonomical relations. Moreover, proposed framework has capability to perform large-scale knowledge extraction from biomedical scientific literature, by using proposed NLP and knowledge-based approaches.
2. Unlike existing approaches [23–26] that generate collection of concepts, properties, and the relations, the proposed framework generates well-defined formal ontology that has inference capability to create new knowledge from existing one.

## Methods

**Fig. 1:**
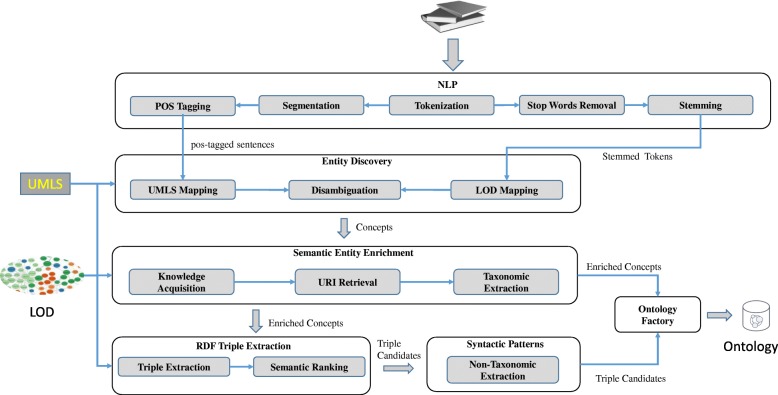
Illustration of framework LOD-ABOG Architecture

Our methodology for automated ontology generation from biomedical literatures is graphically depicted in Fig. 1. A concise description of all LOD-ABOG modules is given in Table 2.

**Table 2:** The main modules of LOD-ABOG

Module Name	Functionality
NLP	Performs the linguistic analysis tasks such as tokenization, segmentation, Part-of-Speech (POS) [62], etc. that is required as input by subsequent modules.
Entity Discovery	Identifies biomedical concepts from free-form text by UMLS and LOD authentication
Semantic Entity Enrichment	Identifies biomedical concepts from free-form text using UMLS and LOD
RDF Triple Extraction	Extracts well-defined information and URIs, as well as taxonomic relations to enrich discovered concepts using LOD.
Syntactic Patterns	Extracts non-taxonomic relations by identifying triples within a sentence that match predefined patterns of words against the input
Ontology Factory	Generates the ontology with respect to RDF, RDFS, OWL and SKOS schemas.

### NLP module

NLP module aims to analyze, interpret and manipulate human language for the purpose of achieving human-like language processing. The input of NLP module is unstructured biomedical literature taken from MEDLINE*/PubMed* [38] *resources.* The NLP module of LOD-ABOG framework uses Stanford NLP APIs [39] to work out the grammatical structure of sentences and perform tokenization, segmentation, stemming, stop words removal, and part-of-speech tagging (POS). Algorithm 1 -Text processing shows the pseudo code of NLP module. Segmentation is the task of recognizing the boundaries of sentences (line 3), whereas *part*-of-*speech tagging* is the process of assigning unambiguous lexical categories to each word (line 4). Tokenization is the process that splits the artifacts into tokens (line 5) while stemming [40] is the process of converting or removing inflected form to a common word form (line 6). For example, ‘jumped’ and ‘jumps’ are changed to root term ‘jump’. Stop words removal is the process of removing the most common words such as “a” and “the” (line 6).

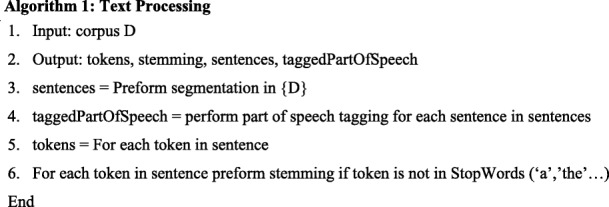



### Entity discovery module

Entity Discovery module is one of the main building blocks of our proposed framework. The main tasks of the entity discovery module are identifying the biomedical concepts within free text, applying n-gram, and performing concepts disambiguation. Identifying biomedical concepts is a challenging task that we overcome by mapping every entity or compound entities to UMLS concepts and LOD classes. Algorithm 2 entity detection shows the pseudo code for entity discovery module. To implement the mapping between entities and UMLS concept ID, we use MetaMap API [41] that presents a knowledge intensive approach based on computational linguistic techniques (lines 3–5). To perform the mapping between entities and LOD classes, algorithm 2 performs three steps; a) it excludes stop words and verbs from the sentence (line 6), b) it identifies multi-words entities (e.g. diabetes mellitus, intracranial aneurysm) using n-gram [42] method with a window size in range of unigram and eight-grams (line 7), c) After that it queries LOD using owl:class, and skos:concept predicates (lines 9–13) to identify concepts . For example, algorithm 2 considers Antiandrogenic as a concept, if there is a triple in the LOD such as the triple “bio: Antiandrogenic rdf:type owl:Class” or “bio: Antiandrogenic rdf:type skos:Concept”, where bio: is the namespace of the relevant ontology. Our detailed analysis shows that using UMLS and LOD (LLD or BioPortal) as a hybrid solution increases the precision and recall of entity discovery. However, using LOD to discover concepts has a co-reference [43] problem that occurs when a single URI identifies more than one resource. For example, many URIs in LOD are used for identifying a single author where, in fact, there are many people with the same name. In biomedical domain ‘common cold’ concept can be related to weather or disease. Therefore, we apply concept disambiguation for identifying the correct resource by using adaptive Lesk algorithm [44] for semantic relatedness between *concepts (lines 15–17)*. Basically, we use the definition of the concept to measure the overlap with other discovered concepts definitions within the text, then we select the concepts that meet the threshold and have high overlap.

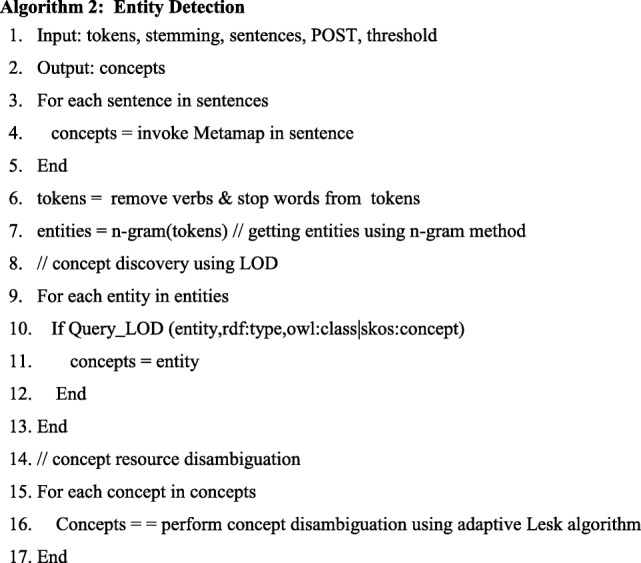



### Semantic entity enrichment module

**Fig. 2:**
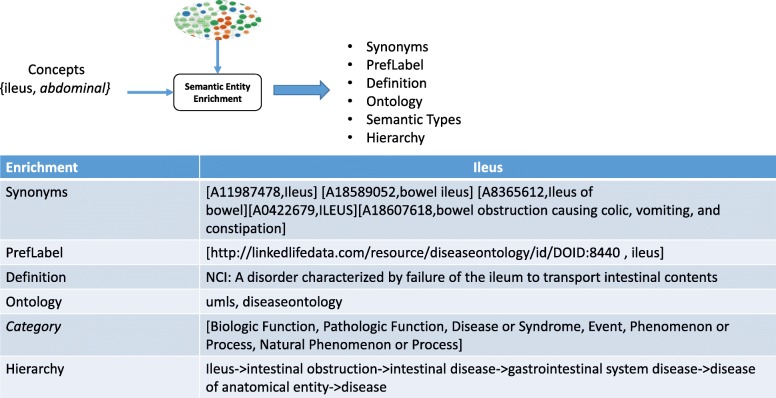
An example of semantic entity enrichment output

For the purpose of improving semantic interoperability in ontology generation, the semantic enrichment module aims to automatically enrich concepts (and implicitly the related resources) with formal semantics by associating them to relevant concepts defined in LOD. Semantic Entity Enrichment module reads all discovered concepts by entity discovery module and enriches each of them with additional, well-defined information which can be processed by machines. An example of semantic entity enrichment output is given in Fig. 2, and algorithm 3 shows pseudo code for Semantic Entity Enrichment Module.

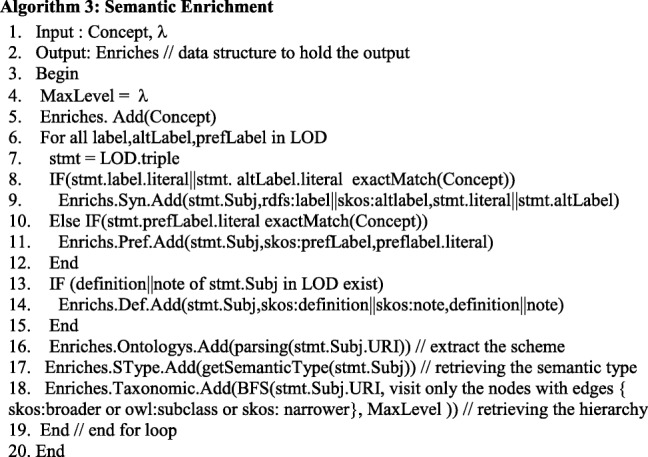



The proposed enrichment process is summarized as follows:

1. Algorithm 3 takes a concept extracted using algorithm 2 and λ (maximum level of ancestors in graph) as input (line 1)
2. For each triple in LOD with predicate (label, altlabel, preflabel) (lines 6–19).
  2.1. Apply exact matching (input concept, value of the predicate) (lines 8–12)
    2.1.1. extract the triple as ‘altlabel or/and preflabel ’
  2.2. Retrieve the definition of the concept from LOD by querying skos:definition and skos:note for the preferable resource (lines 13–15)
  2.3. Identify the concept schema that the concept has been defined in by analyzing URIs (line 16).
  2.4. Acquire the semantic type of a concept by mapping it to UMLS semantic type. Since a concept might map to more than one semantic type, we consider all of them (line 17).
  2.5. Acquire the hierarchy of a concept which is a challenging task. In our proposed framework, we use a graph algorithm since we consider LOD as a large directed graph. Breadth-First Search is used to traverse the nodes that have skos:broader or owl:subclass or skos: narrower edge. This implementation allows multi-level hierarchy to be controlled by input λ (line 18).

### RDF triple extraction module

The main goal of RDF Triple Extraction module is to identify the well-defined triple in LOD that represents a relation between two concepts within the input biomedical text. Our proposed approach provides a unique solution using graph method for RDF triples mining, measures the relatedness of existing triples in LOD, as well as generates triple candidates. Algorithm 4 shows the pseudo code for RDF Triple Extraction.

In our proposed Algorithm 4 Triple Extraction, the depth of BreadthFirstSearch graph call is configurable and provides scalability and efficiency at the same time. We set the depth to optimal value 5 in line 4 for best results and performance. Line 5 retrieves all triples that describe the source input concept using BreadthFirstSearch algorithm. Algorithm 4 only considers the triples that represent two different concepts. The code in lines 7–18 measures the relatedness by matching labels, synonyms, overlapping definitions, and overlapping hierarchy. To enhance the triple extraction as much as possible, we set the matching threshold to 70% (Algorithm 4 lines 13, 15, & 17) to remove the noise of triples in our evaluation. More details on the depth and threshold values are provided in the Discussion section later.

In addition, the module has a subtask that semantically ranks URIs for a given concept by using our algorithm URI_Ranking. The URIs are retrieved from LOD by either the label or altlabel of a resource match. For example, the resource http://linkedlifedata.com/resource/diseaseontology/id/DOID:8440 diseaseontology/id/DOID:8440 is retrieved for the given concept “ileus”. One of the main challenges of retrieving URIs is when one concept can be represented by multiple URIs. For example, concept “ileus” can be represented by more than one as illustrated in Table 3.

**Table 3:** URIs that represent concept “Ileus”

URI1= http://linkedlifedata.com/resource/umls/id/C1258215	
URI2= http://linkedlifedata.com/resource/pubmed/mesh/Ileus	
URI3= http://linkedlifedata.com/resource/phenotype/id/HP:0002595	
URI4= http://linkedlifedata.com/resource/rxnorm/id/1026920	
URI5= http://linkedlifedata.com/resource/diseaseontology/id/DOID:8440	
URI6= http://linkedlifedata.com/resource/umls/id/C0030446	
URI7= http://linkedlifedata.com/resource/diseaseontology/id/DOID:8442	

To resolve this issue, we present algorithm URI_Ranking for ranking the URIs of each concept based on their semantic relatedness. More precisely, for a given concept, the goal is to generate a URI ranking, whereby each URI is assigned a positive real value, from which an ordinal ranking can be used if desired. In a simple form, our algorithm URI_Ranking assigns a numerical weighting to each URI where it first builds for each, a feature vector that contains UMLS semantic type and group type [45–47]. Then it measures the average cosine relatedness between the vectors of every two of those URIs that are relevant to the same concept as written below in algorithm 5. Finally, it sorts them based on their numerical weighting.

### Syntactic patterns module

**Fig. 3:**
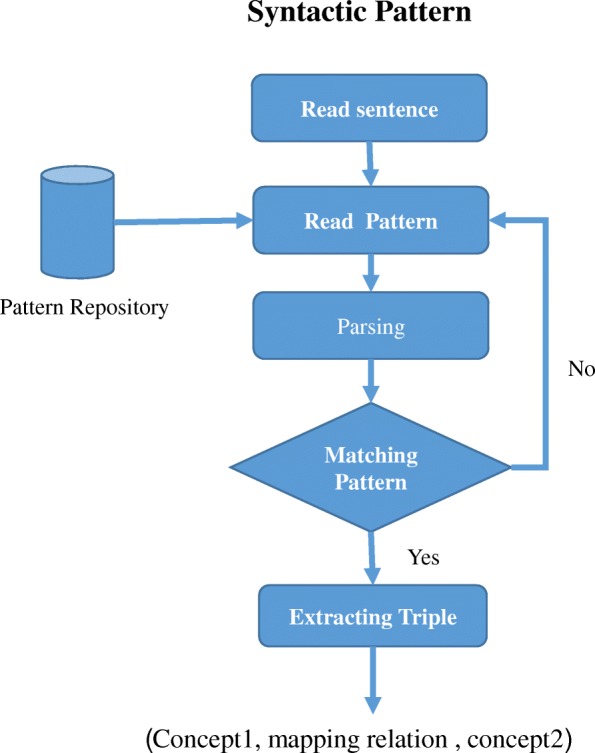
Syntactic Patterns Module Workflow

In our proposed approach, Syntactic Patterns module performs pattern recognition to find a relation between two concepts within a free text which is graphically depicted in Fig. 3. The pattern repository is built by extracting all biomedical patterns with their observer relation from Freepal [48]. After that we ask an expert to map the obtained patterns with their observer relations to health-lifesci vocabulary [49]. In Table 4 we present a sample of patterns and their corresponding observed relations and mapping predicates. In the next stage, we develop an algorithm that reads a sentence, loops through all patterns, applies parsing, and then transforms the matched pattern into a triple candidate. This algorithm takes advantage of semantic enrichment information. For example, if the pattern does not match any discovered concepts within the sentence then the concept synonym is used. This leads to an increase in the recall result. It is important to point out that the algorithm is not case sensitive.

**Table 4:** Patterns and their corresponding observed relations and mapping predicates

Pattern	Observed Relations in Freepal	Predicates in lifesci
[X] causes by [Y]	ns:medicine.disease.causes	http://schema.org/causeOf
[X] disability [Y]	ns:medicine.symptom.symptom_of	http://schema.org/signOrSymptom
[X] treatment of [Y]	treatrel.used_to_treat	http://schema.org/possibleTreatment
[X] drug treatment [Y]	treatrel.used_to_treat	http://schema.org/possibleTreatment
[X] cancer [Y]	ns:medicine.risk_factor.diseases	http://schema.org/diagnosis
example of [X] include [Y]	s:medicine.drug_class.drugs	http://schema.org/drug

### Ontology factory

**Fig. 4:**
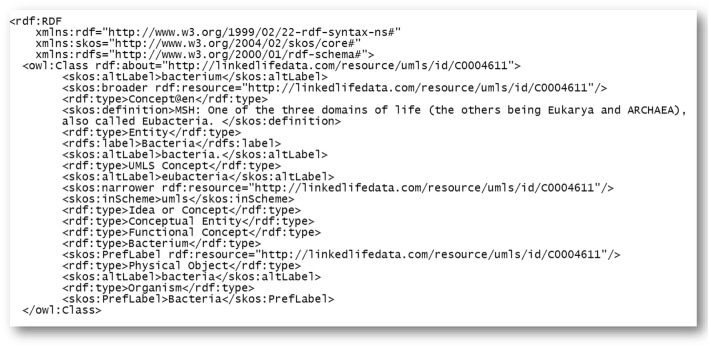
A simplified partial example of ontology generated by LOD-ABOG

This module plays a central role in our proposed framework where it automates the process of encoding the semantic enrichment information and triples candidates to ontology using an ontology language such as RDF, RDFS, OWL, and SKOS. We selected W3C specifications ontologies over the Open Biomedical Ontologies (OBO) format because they provide well-defined standards for semantic web that expedite ontology development and maintenance. Furthermore, they support the inference of complex properties based on rule-based engines. An example of ontology generated by our proposed framework is given in Fig. 4.

In the context of ontology factory, two inputs are needed to generate classes, properties, is-a relations, and association relations. These two inputs are: 1) concepts semantic enrichment from semantic enrichment module and 2) triple candidates from RDF triple extraction and syntactic patterns modules. There are many relations that can be generated using semantic enrichment information. Initially, domain-specific root classes are defined by simply declaring a named class using the obtained concepts. A class identifier (a URI reference) is defined for each obtained class using the top ranked URI that represents the concept. After defining the class of each obtained concept, the other semantic relations are defined. For example, the concepts can have super-concept and sub-concepts, providing property rdfs:subClassof that can be defined using the obtained hierarchy relations. In addition, if the concepts have synonyms then they are given an equivalence defined axiom, “preflabel” property is given for obtained preferable concept and “inscheme” property is given for obtained scheme. Few examples of generated relations from LOD-ABOG are given in Table 5.

**Table 5:** LOD-ABOG Ontology Relations

Semantic Enrichment/Triple Candidate	Ontology Relation
Concept	owl:class
Synonym	owl:equivalentClass, skos:altLabel
PrefLabel	skos:prefLabel
Is-a	rdfs:subClassOf
Concept scheme resource	skos:inScheme
High ranked URI	rdf:ID
Most high ranked URIs	owl:sameAs
Semantic type	rdf:type
Definition	skos:definition

### Evaluation

Our proposed approach offers a novel, simple, and concise framework that is driven by LOD. We have used three different ontology evolution approaches [50] to evaluate our automated ontology generation framework. First, we develop and experimentally apply our automated biomedical ontology generation algorithms to evaluate our framework based on Task-based Evaluation [51, 52] using CDR corpus [53] and SemMedDB [54]. Second, we have done baseline ontology-based evaluation using Alzheimer’s disease ontology [55] as gold standard. Third, we compared our proposed framework with one of the state of the art ontology-learning frameworks called “OntoGain”. We use Apache Jena framework [56] which is a development environment that provides a rich set of interactive tools and we conduct experiments by using 4-core Intel(R) Core(TM) **i7**-**4810MQ** CPU @ 2.**80 GHz** and 64 bits Java **JVM**. Furthermore, during our evaluation, we found an entity can consist of a single concept word or a multi-word concept. Therefore, we considered only the long concept match and ignored the short concept to increase the precision. In addition, we found a limitation where all entities cannot be mapped to UMLS concept ID due to the large volume of entities and abbreviations in biomedical literature and its dynamic nature given that new entities are discovered every day. For example, the entity “Antiandrogenic” has no concept ID in UMLS. To resolve it we considered LOD-based technique. Also, we applied different window sizes ranging from 1 to 8 as input for n-gram method. However, we found that window size equal to 4 was optimal as the other values decrease the entity detection module performance, recall yielded a very low value, and an average precision when window size was less than 4. On the other hand, recall increased when window size was greater than 4 but precision was very low.

### The dataset

For task base evaluation, first we employ CDR Corpus [53] titles as input and as gold standard for entity discovery evaluation: the annotated CDR corpus contains 1500 PubMed titles of chemicals, diseases, and chemical-induced disease relationships where Medical Subject Headings 2017 (Mesh Synonym) [57] has been used as gold standard for synonym extraction evaluation. Furthermore, we manually build gold standard for broader hierarchy relation for all discovered concepts from CDR using Disease Ontology (DO) [58] and Chemical Entities of Biological Interest (ChEBI) [59]. On the other hand, we use relations between DISEASE/TREATMENT entities data set as the gold standard for non-hierarchy relation discovery evaluation [60].

Next, for task base evaluation, we downloaded Semantic MEDLINE Database (SemMedDB) ver 31, December 2017, release [54], which is a repository of biomedical semantic predications that extracted from MEDLINE abstracts by the NLP program SemRep [61]. We constructed benchmark dataset from SemMedDB. The dataset consists of 50,000 sentences that represent all relation types that exist in SemMedDB. Furthermore, we extracted all semantic predications and entities for each sentence from SemMedDB and used them as benchmark for relation extraction and concept extraction evaluation, respectively.

For baseline ontology evaluation, we selected 40,000 titles that relevant to the “Alzheimer” domain from MEDLINE citations published between Jan-2017 to April-2018. Furthermore, we have extracted a subgraph of Alzheimer’s disease Ontology. The process of extracting subgraph out of the Alzheimer’s Disease Ontology was done using following steps: a) we downloaded the complete Alzheimer’s Disease Ontology from Bioportal as an OWL file, b) uploaded the OWL file as model graph using Jena APIs, c) retrieved the concepts that match to the entity “Alzheimer”, d) retrieved properties (synonyms), and relations for the extracted concepts in step c. This resultant subgraph contained 500 concepts, 1420 relations, and 500 properties (synonyms).

## Results

To evaluate our proposed entity-discovery ability to classify concepts mentioned in context, we annotate the CDR corpus titles of chemicals and diseases. In this evaluation, we use precision, recall, and F-measure as evaluation parameters. Precision is the ratio of the number of true positive concepts annotated over the total number of concepts annotated as in Eq. (1), whereas, recall is the ratio of the number of true positive concepts annotated over the total number of true positive concepts in gold standard set as in Eq. (2). F-measure is the harmonic mean of precision and recall as in Eq. (3). Table 6 compares the precision, recall, and F-measure of MetaMap, LOD, and the hybrid method.

**Table 6:** Comparison of different methods for concepts discovery

Method	Concepts Discovery		
	Recall %	Precision %	F-Measure %
UMLS	63.12	22.53	33.20
LOD	77.01	23.36	35.84
UMLS + LOD	81.13	45.29	58.12

**Fig. 5:**
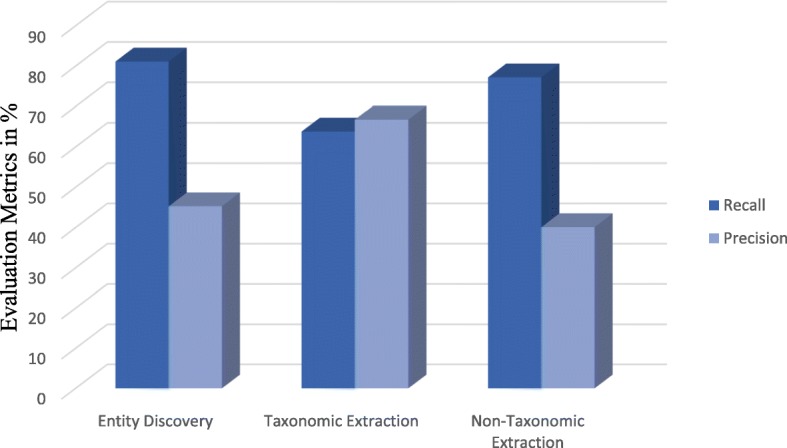
Results Evaluation of the primary ontology generation tasks in LOD-ABOG

**Fig. 6:**
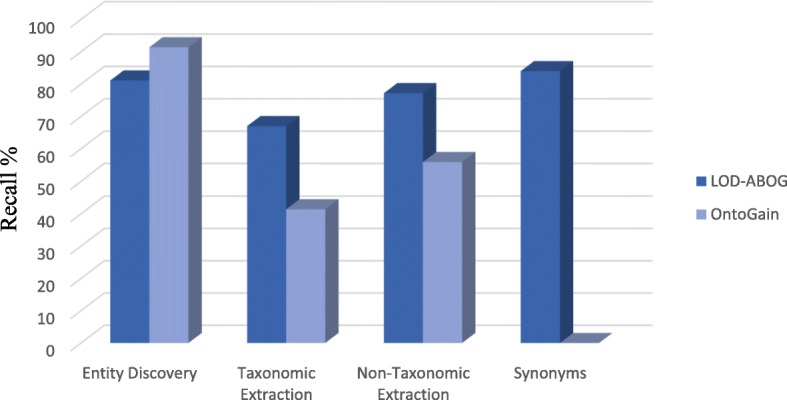
Comparison of Recall between LOD-ABOG and OntoGain Framework

**Fig. 7:**
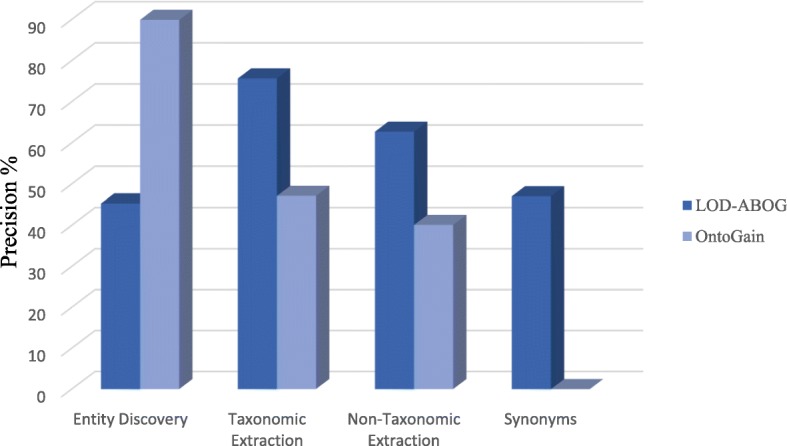
Comparison of Precision between LOD-ABOG and OntoGain Framework

The evaluation results of hierarchy extraction was measured using recall as in Eq. (4), precision as in Eq. (5), and F-measure as in Eq. (3). In addition, the evaluation result of non-hierarchy extraction was measured using recall as in Eq. (6), precision as in Eq. (7), and F-measure again as Eq. (3). Table 7 compares the precision, recall, and F-measure of hierarchy extraction, while Table 8 compares the precision, recall, and F-measure of non-hierarchy extraction. The results of the main ontology generation tasks are graphically depicted in Fig. 5. Nevertheless, we assessed our proposed framework with one of the state of the art ontology acquisition tools: namely, OntoGain. We selected OntoGain tools because it is one of the latest tools, that has been evaluated using the medical domain and the output result is in OWL. Figures 6 and 7 depict the comparison between our proposed framework and OntoGain tools using recall and precision measurement. These figures provide an indication of the effectiveness of LOD in ontology generation.

1 $$ \mathbf{Concept}\ \mathbf{Precision}=\frac{\mathrm{correct}\ \mathrm{retrieved}\ \mathrm{Concepts}}{\mathrm{total}\ \mathrm{retrieved}\ \mathrm{Concepts}\ } $$

2 $$ \mathbf{Concept}\ \mathbf{Recall}=2\times \frac{\mathrm{correct}\ \mathrm{retrieved}\ \mathrm{Concepts}}{\mathrm{total}\ \mathrm{correct}\ \mathrm{concepts}} $$

3 $$ \mathbf{F}-\mathbf{measure}=2\times \frac{precision\ x\  recall}{precision+ recall} $$

4 $$ \mathbf{Hierarchy}\ \mathbf{Recall}=\frac{old\  standard\cap Hierarachy\ extracted\ }{Gold\ standard} $$

5 $$ \mathbf{Hierarchy}\ \mathbf{Precision}=\frac{\  Gold\ standard\cap Hierarachy\ extracted}{Hierarachy\ extracted} $$

6 $$ \mathbf{Non}-\mathbf{Hierarchy}\ \mathbf{Recall}=\frac{Gold\ standard\cap Non- Hierarachy\ extracted\ }{old\  standard} $$

7 $$ \mathbf{Non}-\mathbf{Hierarchy}\ \mathbf{Precision}=\frac{Gold\ standard\cap Non- Hierarachy\ extracted}{Hierarachy\ extracted} $$

**Table 7:** Evaluation of hierarchy extraction results

Hierarchical Relation Extraction			
	Recall %	Precision %	F-Measure %
Disease Concepts	77.44	80.11	78.75
Chemical Concepts	50.20	53.43	51.76
Disease + Chemical Concepts	63.82	66.77	65.26

**Table 8:** Evaluation of non-hierarchy extraction results

Non-Hierarchical Relation Extraction		
Recall %	Precision %	F-Measure %
77.20	40.1	52.78

Moreover, we compared the generated ontology from the proposed framework to Alzheimer’s disease ontology that has been constructed by domain expert [55]. Table 9 compares results of our ontology generation to Alzheimer’s disease Ontology. The results indicate F-measure of 72.48% for concepts detection, 76.27% for relation extraction, and 83.28% for property extraction. This shows satisfactory performance of the proposed framework; however, the F-measure could be improved further by domain expert during verification phase. Table 10 compares our concept and relation extraction results against SemMedDB.

**Table 9:** Comparison of results with baseline ontology (Alzheimer ontology)

Extraction	Recall %	Precision %	F-measure %
Concepts	87.28	62.50	72.48
Relations	77.47	75.12	76.27
Properties	87.21	79.68	83.28

**Table 10:** Comparison of results with SemMedDB

Extraction	Recall %	Precision %	F-Measure %
concepts	89.34	75.23	81.68
Hierarchy relations	82.64	72.86	77.44
Non-Hierarchy relations	45.25	81.25	58.12

## Discussion

Our deep dive analysis shows the effectiveness of LOD in automated ontology generation. In addition, re-use of the crafted ontologies will improve the accuracy and quality of the ontology generation. All of these measures address some of the shortcomings of existent ontology generation. Moreover, the evaluation results in Table 6 show that our concept discovery approach performs very well and matches the results reported in the literature. However, the evaluation results in Figs. 6 and 7 shows OntoGain outperforms our concept discovery approach. Whereas OntoGain considers only multi-word concepts in computing precision and recall, our approach considers both multi-word terms and single-word terms. In the hierarchical extraction task, our hierarchy extraction has significant improvement results than OntoGain. Likewise, our syntactic patterns approach on non-taxonomic extraction delivers better results in comparison to OntoGain. In Algorithm 4, we used a threshold parameter δ to increase the accuracy of extracting non-hierarchy relations. We found that setting δ to low value generated a lot of noise relations, whereas increasing it generated better accuracy. However, setting δ to a value higher than 70% yielded a lower recall. Also, we used the depth parameter γ to control the depth of knowledge extraction from LOD. We observed a lesser degree domain coverage when γ is in range [1, 2], but the coverage gradually improved when γ is in range [3, 5]. Nevertheless, when γ> 5 then noise data increased so rapidly. Though the relations defined in the ontology are limited; for example, the disease ontology only defines the hierarchy relations, but very few of the non-hierarchy relations are defined. This is like most existent ontologies which do not define constraints such as rdfs:domain, which helps improve the ability of an ontology extraction system to make accurate inferences. Despite the benefits brought by Linked Open Data, its use in the industrial internet and healthcare sector has not been fully welcomed due to some of its performance issues. To correct its flaws, we proposed a graph-traversal approach using breadth first search, which leads to improve the speed of moving from one node to another without writing very complex queries. As shown in Table 10, the concept extraction and hierarchy relation extraction tasks are competitive in comparison to SemMedDB. However, the non-hierarchy extraction shows low recall due to the syntactic pattern limitation, therefore improving the non-hierarchy extraction is part of our future works.

Furthermore, the precision and recall of our proposed framework could be further improved by domain experts during the verification phase. The results are encouraging and show that we can downsize the requirement for intensive labor. In addition, the framework will enable experts to enforce ontology engineering in a more efficient and effective way.

## Conclusion

Ontology is the cornerstone of the semantic web vision. In addition, it provides a common and shared understanding about concepts in a specific domain, reuse domain knowledge, and data interoperability. However, the manual ontology construction is a complex task and is very time consuming. Therefore, we presented a fully automated ontology generation framework that is empowered by biomedical Linked Open Data, integrates natural language processing, syntactic pattern, graph algorithms, semantic ranking algorithms, semantic enrichment, and RDF triples mining to make automatic large-scale machine processing possible, minimize and downsize requirements and complexity, and improve the accuracy of ontology generation. Ontology is not used only for better search, data interoperability, and presentation of content, but more importantly it represents the foundation of future innovative ways to manage dormant content assets and transform the Web of document to Web of Data.

### Future work

Our future work includes an extension of the framework to support non-biomedical domain ontology generation. In addition, we plan to integrate machine learning and repository of semantic predications (SemMedDB) to the framework to further improve F-measure of concepts and non-hierarchy relations extractions.

## Abbreviations

BioPortal: repository of biomedical ontologies
LLD: Linked Life Data
LOD: Linked Open Data
LOD-ABOG: Linked Open Data-Based Framework for Automated Biomedical Ontology Generation;
OBO: Open Biomedical Ontologies
OWL: Web Ontology Language
RDF: Resource Description Framework
RDFs: Resource Description Framework Schema
SKOS: Simple Knowledge Organization System
UMLS: Medical Language System
